# Pressure Self-focusing Effect and Novel Methods for Increasing the Maximum Pressure in Traditional and Rotational Diamond Anvil Cells

**DOI:** 10.1038/srep45461

**Published:** 2017-04-21

**Authors:** Biao Feng, Valery I. Levitas

**Affiliations:** 1 Department of Aerospace Engineering, Iowa State University, Ames, Iowa 50011, USA; 2 Theoretical Division, Los Alamos National Laboratory, Los Alamos, New Mexico 87545, USA; 3 Departments of Aerospace Engineering, Mechanical Engineering, and Material Science and Engineering, Iowa State University, Ames, Iowa 50011, USA; 4 Ames Laboratory, Division of Materials Science and Engineering, Ames, Iowa 50011, USA

## Abstract

The main principles of producing a region near the center of a sample, compressed in a diamond anvil cell (DAC), with a very high pressure gradient and, consequently, with high pressure are predicted theoretically. The revealed phenomenon of generating extremely high pressure gradient is called the pressure self-focusing effect. Initial analytical predictions utilized generalization of a simplified equilibrium equation. Then, the results are refined using our recent advanced model for elastoplastic material under high pressures in finite element method (FEM) simulations. The main points in producing the pressure self-focusing effect are to use beveled anvils and reach a very thin sample thickness at the center. We find that the superposition of torsion in a rotational DAC (RDAC) offers drastic enhancement of the pressure self-focusing effect and allows one to reach the same pressure under a much lower force and deformation of anvils.

The highest possible static pressure is currently produced by compressing a thin sample/gasket by two diamond anvils in DAC^1^,^2^,^3^ (Fig. 1). One of the goals in high pressure research is to reach the highest possible pressure to explore material behaviors and find unknown phases at extreme conditions. Another objective is to achieve medium-high pressure without breaking diamonds, so they can be reused multiple times. Despite the significant progress in modeling stress-strain states of samples and anvils using FEM^4^,^5^,^6^,^7^,^8^,^9^,^10^,^11^ and optimization of geometry and loading conditions^5^,^7^,^9^,^10^,^11^,^12^, strict formulation of the optimization problem is lacking. Qualitatively, experimental results for achieving the record pressures^2^,^3^ hint that smaller region of diamond under the highest pressure indicates higher anvil strengths. Indeed, in this case, there is less probability of finding a defect causing fracture of the diamond anvil in a highly stressed region. Also, for a relatively low pressure gradient along the radius of a sample, the resultant force applied on the anvils is large. This causes bending of an anvil and the cup-like shape of a culet (the cupping phenomenon^1^,^7^,^10^). The cupping of an anvil impedes a sample’s radial plastic flow. That is why our *goal* is to theoretically find the main principles of producing a region near the center of a sample with a very high pressure gradient (which we call the pressure self-focusing effect) and, consequently, with high pressure. We first generalize a well-known simplified equilibrium equation^13^,^14^,^15^ and make our main predictions analytically. Then, we use our recently developed advanced model and FEM simulations^8^ (which give good correspondence with experiments in ref. 1) to check and further refine the main reasons for the pressure self-focusing effect. Finally, we solve the problem on compression and torsion of a sample in RDAC (Fig. 1) and demonstrate that torsion offers drastic enhancement of the pressure self-focusing effect and allows us to reach the same pressure in DAC under much lower force and deformation of anvils. Consequently, a method for achieving record pressures is related to applying compression and torsion in RDAC with beveled anvils and reaching a small sample thickness.

## Analytical treatment

By considering equilibrium in the radial direction *r* of an elemental volume of a sample in the deformed configuration in DAC (see Appendix), we obtain the following equation


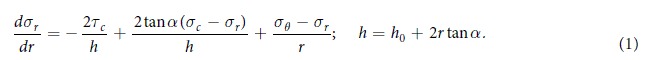


**Figure 1:**
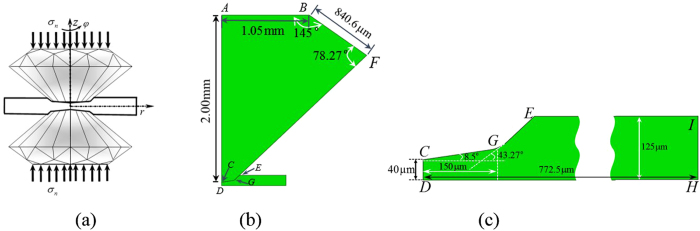
(**a**) Rotational diamond anvil cell scheme, (**b**) a quarter of the sample and anvil in the initial undeformed state and the geometry of anvil, and (**c**) the geometry of sample in the undeformed state.

Here, *σ*_*r*_ and *σ*_*θ*_ are the normal radial and hoop stresses averaged over the sample thickness; *τ*_*c*_ and *σ*_*c*_ are the local friction and normal contact stress at the contact surface between the sample and anvil; *h* is the current sample height and *h*_0_ is the current height at *r* = 0; and *α* is the angle between the contact surface and the *r* axis. For an intense plastic flow, the friction stress at the contact surface reaches the yield strength in shear 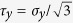 (where *σ*_*y*_ is the yield strength in compression), which leads to *σ*_*z*_ = *σ*_*r*_ = *σ*_*θ*_ = *p* at the contact surface due to the von Mises yield condition. Since the sample is thin and the variation of normal stress along the thickness is small^8^, we obtain *σ*_*z*_ ≈ *σ*_*r*_ ≈ *σ*_*θ*_ ≈ *p*. If *α* is a small angle (such as *α* = 8.5°) in Fig. 1, then *σ*_*c*_ ≈ *σ*_*z*_ ≈ *σ*_*r*_ and we obtain a simplified equilibrium equation


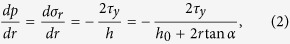


which was used in previous work^13^,^14^,^15^,^16^. We show that additional terms in Eq. (1) are important for our purpose. The yield strength *σ*_*y*_ is linearly dependent of pressure *p*^8^,^11^,^12^,^14^,^16^


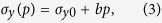


where 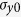 is the yield strength at *p* = 0, and *b* is a parameter. For *α* = 0 and *b* = 0, the pressure gradient in Eq. (2) is constant along the radial direction. It is clear from Eq. (2) that a large pressure gradient can be obtained at the center (*r* ≈ 0) for a small *h*_0_. A larger angle *α* leads to a larger increase in pressure gradient toward the center. However, the maximum *α* is limited by fracture of diamond at relatively low pressure^10^, and we use the typical experimental value *α* = 8.5° (see Fig. 1).

Despite the approximate model, it leads to a simple analytical prediction of the pressure self-focusing effect, which is confirmed and elaborated below by much more sophisticated numerical simulations.

## Numerical Results and Discussion

We use a model of an isotropic, elastoplastic material with large elastic and plastic deformations, which is formulated in ref. 8 and presented in the Appendix. It aptly reproduces experimental pressure distribution curves and deformation of an anvil (cupping) from ref. 1 at pressures up to 300 GPa^8^. The Murnaghan elastic potential and J_2_ plasticity theory are used with the pressure-dependent yield strength in Eq. (3). A diamond anvil with cubic lattice symmetry is subjected to a finite elastic strain and obeys a nonlinear elastic rule. This model and corresponding algorithms^8^ are implemented in the FEM code ABAQUS. At the contact surface between diamond and sample, the Coulomb friction model is applied. Simulations are performed for a rhenium sample, which is one of the most popular gasket metals^1^,^7^,^16^. For rhenium, *σ*_*y*0_ = 8.00 GPa and *b* = 0.04^8^,^16^, but we also consider *b* = 0 to elucidate the effect of pressure-dependence of the yield strength.

A schematic of a RDAC and the geometry of the diamond and sample are shown in Fig. 1. Without the rotation of an anvil, the geometry and load are considered to be axisymmetric (and therefore correspond to traditional DAC) when a normal stress *σ*_*n*_ in Fig. 1(a) is applied on the anvils. For RDAC, the loading is 3D. This is shown in Fig. 1(a): an axial compressive normal stress *σ*_*n*_ is applied first, and then, the torque rotates one anvil with respect to another by an angle *φ* under a fixed *σ*_*n*_. In our simulations, 100 quadrilateral 4-node bilinear elements were assigned along the half-thickness. The results did not change when we used 50 elements.

### Pressure self-focusing effect in DAC with rigid diamond

**Figure 2:**
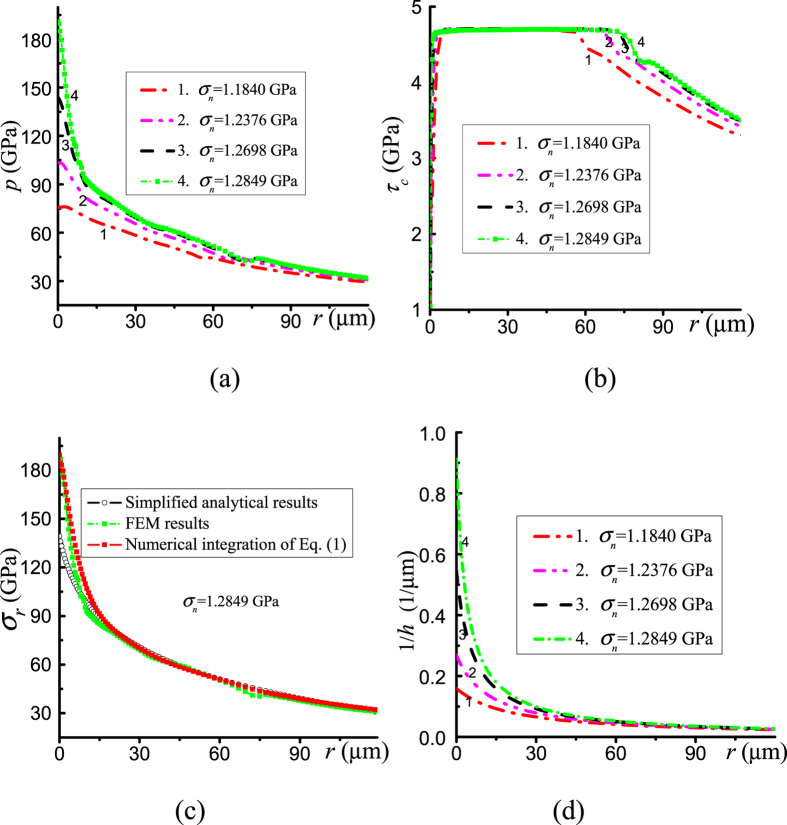
Distributions of (**a**) pressure *p*, (**b**) friction stress *τ*_*c*_, and (**c**) radial stress *σ*_*r*_ at the contact surface, and (**d**) the inversed thickness 1/*h* for the sample with a constant yield strength for different applied normal stress *σ*_*n*_.

To separate the effect of pressure-dependence of the yield strength, we start with the simplest case, *b* = 0, and also consider the rigid diamond anvils. Integrating Eq. (2) with an initial condition that for *r* = *r*_1_, the radial stress *σ*_*r*_ = *σ*_*r*1_, we obtain


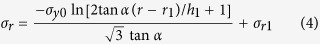


where *h*_1_is the thickness of the sample at *r* = *r*_1_. The FEM results on the pressure distribution at the contact surface are shown in Fig. 2(a). With an increase in the applied stress *σ*_*n*_, the pressure gradient increases much more significantly at the center of a sample than at the periphery. When *σ*_*n*_ increases from 1.2698 to 1.2849 GPa, significant pressure growth is visible only near the center of a sample. Additionally, the pressure gradient is large even at *r* = 0. Such a large pressure gradient at *r* = 0 corresponds to experiments^1^, and it is also described by our model^8^. This offers conceptual experimental proof of our suggestion that a large pressure gradient can be produced by a combination of beveled anvils and a small sample thickness. Friction stress reaches *τ*_*y*_ in a major portion of the contact surface (Fig. 2(b)), which does not grow significantly with an increasing load. The increase in friction stress in the center slightly contributes to a large pressure gradient near the center. Figure 2(d) shows the main reason for the localized large pressure gradient at the center: a strong, localized increase in the inversed thickness 1/*h*, to which pressure gradient is proportional according to Eq. (2).

The simplified analytical curve based on Eq. (4) is consistent with numerical results for *r* ≥ 20 μm, but it is well below the FEM results at the center of the sample. Thus, FEM gives an even stronger pressure self-focusing effect than that expected from the simplified equation, and it also indicates that the last two terms in Eq. (1) can play a significant role. To prove validity of Eq. (1) in the last two terms, we substituted into Eq. (1) the distribution of stresses *σ*_*c*_ and *σ*_*θ*_ from the numerical solution. We then integrated Eq. (1) numerically. Figure 2(c) shows that the FEM results and solution of Eq. (1) are very close. Thus, this advanced Eq. (1) is significantly more precise than the traditional Eq. (2) near the center of sample when a very large pressure gradient is present. Also, Eq. (2) cannot be used for determination of the yield strength of material in the region close to the center, as it was done before^14^,^15^,^16^. Friction stress *τ*_*c*_ obeys the Coulomb rule *τ*_*c*_ = *μσ*_*c*_ in the external low-pressure region until it reaches the yield strength in shear *τ*_*y*_, which is constant for *b* = *0*. For larger pressures, it is substituted with plastic friction *τ*_*c*_ = *τ*_*y*_ (Fig. 2a). Note that Eq. (1) can be approximately extended for larger pressure and deformation of an anvil (cupping). For this purpose, the shape of anvil *h*(*r, σ*_*n*_), which is determined either experimentally or by FEM at different applied stresses *σ*_*n*_, could be interpolated and used in Eq. (1).

**Figure 3:**
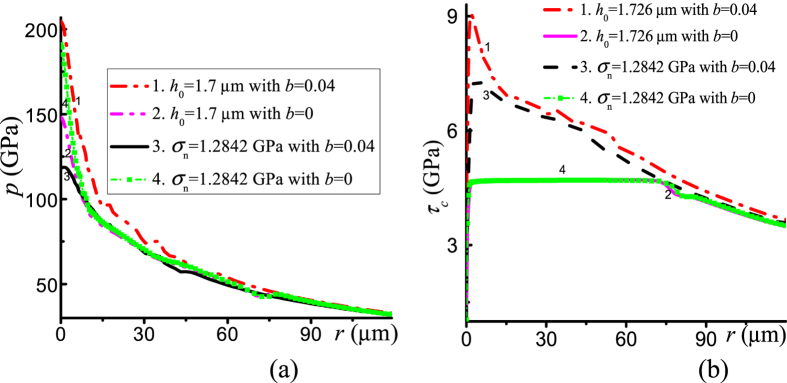
Distributions of (**a**) pressure *p* and (**b**) friction stress *τ*_*c*_ at the contact surface for compression by rigid diamonds. The yield strength is constant (*i.e*., *b* = 0) for curves (2) and (4) and pressure dependent (*i.e*., *b* = 0.04) for curves (1) and (3). The thickness of sample at the center *h*_0_ is 1.7 μm for curves (1) and (2).

To evaluate the effect of the pressure-dependent yield strength, we compare results for *b* = 0 and *b* = 0.04. According to Fig. 3(b), friction stress is significantly larger for pressure-dependent yield strength, especially at the sample center. This should increase the pressure gradient according to Eq. (2). This is indeed the case when we compare samples of the same thickness, see Fig. 3(a), curves 1 and 2. However, when we compare the cases with the same applied stress*σ*_*n*_ = 1.2842 GPa, the result is opposite: the pressure gradient and pressure at the sample center are significantly larger for the pressure-independent yield strength. The friction stress is smaller for the pressure-independent yield strength (Fig. 3(b)), which should lead to a smaller pressure gradient. However, smaller friction stress intensifies radial flow, which leads to significant reduction in the sample thickness. Indeed, for *σ*_*n*_ = 1.2842 GPa, the sample thickness at the center with pressure-independent yield strength is 1.1 μm versus4.4 μm with pressure-dependent strength. That is why the resultant effect leads to an increase in the pressure gradient for pressure-independent yield strength.

### Pressure self-focusing effect in RDAC

**Figure 4:**
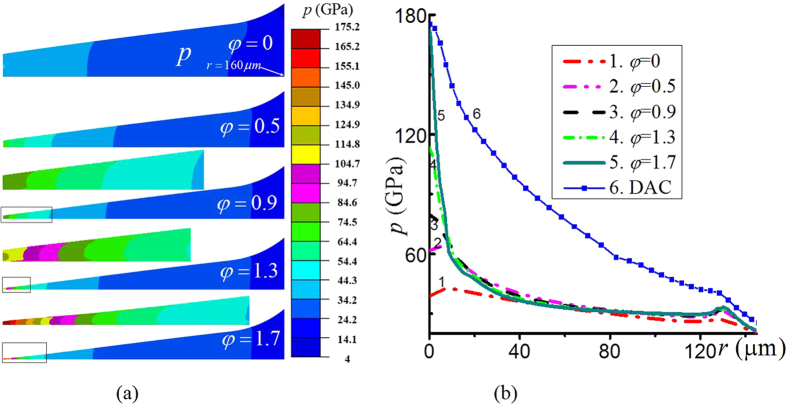
Distribution of the pressure *p* (**a**) in the sample and (**b**) at contact surface under an applied normal stress *σ*_*n*_ = 1.095 GPa with the growing rotation angle (in radians). The zoomed central part of a sample is shown above the sample for each rotation angle. For comparison, curve 6 in Fig. 4 (**b**) is shown for compression in DAC with deformable diamonds to the same pressure at the center.

**Figure 5:**
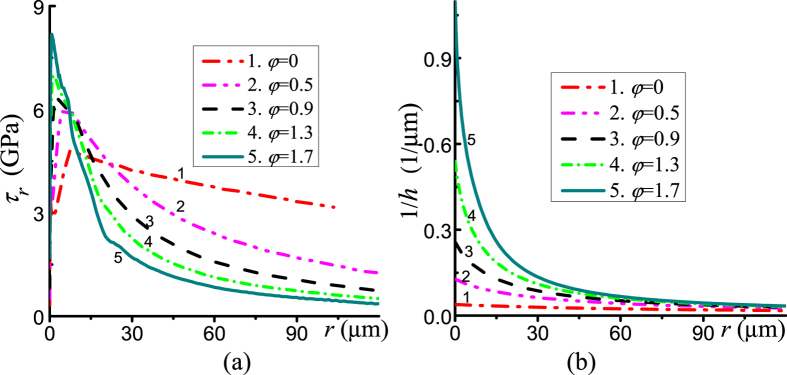
Distributions of (**a**) the radial component of the friction stresses *τ*_*r*_ and (**b**) inverse thickness 1/*h* of sample under a fixed applied normal stress *σ*_*n*_ = 1.095 GPa with the increasing rotation angle *φ*.

We consider the deformable diamond and the sample with pressure-dependent yield strength compressed and twisted in RDAC. Before rotation, the pressure and pressure gradient are small (Fig. 4) due to a small friction stress (Fig. 4) and a large thickness of the sample. During rotation of an anvil, the velocity of relative sliding and friction shear stress are inclined to the radial direction. Also, the radial component *τ*_*r*_ of friction stress is smaller than the total friction stress. With increasing torsion, the torsional component of the shear stress *τ*_*θ*_ increases, and the radial component *τ*_*r*_ decreases in the major outer part of a sample. However, both *τ*_*θ*_ and *τ*_*r*_ increase in the central part due to the increase in pressure. The difference between the radial friction stress *τ*_*r*_ at the center and at the periphery is much larger in the case of RDAC (shown in Fig. 5(a)) than in the case of DAC (shown in Fig. 3(b)). This makes a significant contribution to the difference in pressure at the center and periphery in Fig. 4(b) for RDAC and DAC.

With an increasing rotation angle, the material flows to the outside, which significantly reduces thickness (Figs 4(a) and 5(b)). For example, when *φ* increases from 0 to 1.7, the thickness of the sample reduces from 25.5 to 0.9 μm; the rotation from *φ* = 0.5 to *φ* = 1.7 leads to a decrease of thickness by an order of magnitude. This leads to a gradient in the inverse of the thickness 1/*h* that is much larger than for compression in DAC. For this reason, the pressure gradient in RDAC is much larger than in DAC (Fig. 4(b)). Note that the significant difference between curve 6 in Fig. 4(b) for DAC and the curves in Fig. 3 illustrate the effect of deformation of diamond anvils.

Based on the analysis provided above, we can drastically increase the pressure gradient and pressure at the sample center much more effectively by rotating the diamond anvil under a fixed applied normal stress than by compression in DAC (Fig. 4(b)). For RDAC, pressure grows mostly at the center, as desired, in a much smaller region that in DAC. A much smaller force acting over the culet CG in Fig. 1 causes much less deformation, especially bending, of an anvil. This postpones cupping to a much higher pressure at the center. In addition, there is a much lower probability of finding a defect leading to fracture of diamond within the smaller volume. Note that the obtained results for RDAC vs. DAC are very unexpected since the existing analytical model^17^, FEM simulations with a simplified model^18^, and experiments^6^,^19^ show that pressure distribution during rotation of a flat anvil does not change and coincide with that for DAC below 10 GPa.

**Figure 6:**
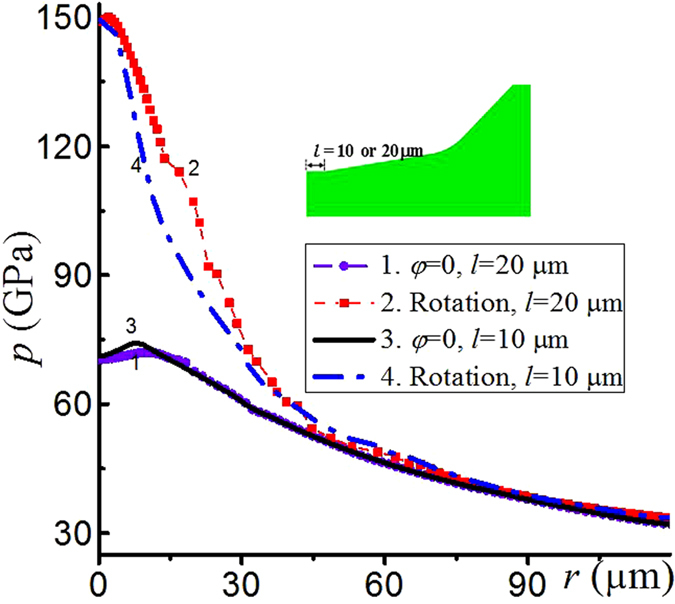
Pressure distribution at the contact surface of the sample with an initially flat part at the center with radius *l* = 10 μm (3, 4) or 20 μm (1, 2) under *σ*_*n*_ = 1.3 GPa. The rotation angle is 0.58 radians for (2) and 0.40 radians for (4), and there is no rotation for (1, 3).

One can control the pressure gradient and the radius of the region in which pressure exceeds the desired value by introducing a flat contact surface at the center (Fig. 6). If the radius of the flat region increases from 0 to *l* = 10 and further to *l* = 20 μm, then for the maximum pressure of 150 GPa at the center, the radius of the region with pressure above 100 GPa increases by factors of 2.5 and 4.3, respectively, in comparison with the case of *l* = 0. During rotation of an anvil, the maximum pressure doubles at rotation angle 0.58 with *l* = 20 μm and 0.40 with *l* = 10 μm. While a flat region reduces pressure gradient, it cannot lead to the quasi-homogenous pressure distribution, which is clear from Eq. (2). If quasi-homogeneous pressure distribution is desirable, one has to use a gasket-sample system and optimize geometric parameters and strength of gasket for different sample materials. Examples of such studies at much lower pressures can be found in refs 20,21.

## Concluding Remarks

To conclude, we theoretically predicted that the pressure self-focusing effect at the center of the sample compressed in DAC can be obtained by compressing the sample to a very small thickness with beveled anvils. For flat anvils, this effect does not exist. The well-known, simplified equilibrium Eq. (2) qualitatively predicts this effect; for quantitative predictions, it was generalized in Eq. (1). The FEM solution with an advanced model^8^ (that reproduces well experimental pressure distribution curves with large pressure gradient and deformation of an anvil (cupping) from ref. 1 at pressures up to 300 GPa) predicts an even stronger pressure self-focusing effect than Eq. (2). The effect of pressure-dependence of the yield strength is dual: it promotes the pressure self-focusing effect for the same sample thickness, but suppresses it for the same applied load. The main problem in further increasing the pressure gradient and, consequently, pressure under compression in DAC is related to elastic deformation and cupping of anvils, which prevent reduction of the sample thickness below a critical value. The main result of the paper is that the superposition of torsion in RDAC drastically enhances the pressure self-focusing effect and allows one to reach the same pressure with much less force and deformation of anvils. This is because rotation of an anvil under constant applied force significantly reduces the sample thickness. In addition, the radial component of the friction stress that determines the pressure gradient strongly increases at the center due to the pressure-dependence of the yield strength. Such a drastic increase in pressure gradient due to rotation of an anvil seems very counterintuitive since it was never reported in experimental literature^15^,^19^,^20^,^22^,^23^,^24^. Moreover, it is well-known from experiments^24^,^25^, our theory^17^, and FEM simulations^18^ that rotation of an anvil under a constant force does not change the pressure distribution for *flat* anvils (if phase transformation does not occur). Here, using the same model, we changed geometric parameters and obtained opposite and completely unexpected results which make our prediction even more nontrivial and attractive.

We expect that experimentalists will be motivated to test our proposal for an increasing pressure gradient and maximum pressure. With any outcome, this will lead to important progress. Even if our predictions contradict the experiments, there will be need for significant advancements in the theory of plastic flow at high pressure and large pressure gradient.

Note that the predicted effect should not be confused with the pressure self-multiplication effect in RDAC during phase transformations observed experimentally^19^,^24^,^25^. It was rationalized by an increase in the yield strength during phase transformations using the analytical model^17^ and FEM simulations^21^,^26^,^27^. This can be used as an additional method to increase the pressure gradient and maximum pressure.

Note that it was shown in ref. 7 for compression in DAC, the friction coefficient *μ* essentially does not affect the stress and strain fields in a sample. We found that this is not the case for torsion in RDAC under a fixed load due to much lower contact stress *σ*_*c*_ and pressure *p* in the major part of the contact surface between the sample and anvil (see Fig. 4). For compression in DAC, due to very high contact stress *σ*_*c*_, *μσ*_*c*_ > *τ*_*y*_ in the major portion of the contact surface, where the Coulomb friction is substituted with complete cohesion. It results in plastic friction condition *τ*_*c*_ = *τ*_*y*_ (Fig. 1(b)). That is why variation of *μ* essentially does not affect the pressure gradient and distribution of pressure both in Eq. (2) and numerical simulations. In contrast, for torsion in RDAC due to much smaller pressure (excluding center of a sample), the Coulomb friction condition is fulfilled at a much larger portion of the contact surface. That is why variation of the Coulomb friction coefficient significantly changes the friction stress. This consequently changes the contact normal stress and the entire stress-strain state. The effects of Coulomb friction and other geometric and material parameters will be considered in detail in a future work.

It is interesting to note in Fig. 2(a) that the increase in applied compressive stress *σ*_*n*_ by only ~0.1 GPa increases the maximum pressure at the center by ~100 GPa. The ratio of the area where *σ*_*n*_ is applied to the culet area is (1050/150)^2^ = 49, thus the averaged pressure over the culet area increases by only ~4.9 GPa. However, pressure increase is extremely heterogeneous. Namely, it is very small at large radii and very large for small radii, which contribute only a small amount to the total area and force. If (just for a rough estimate) pressure would change homogeneously within a radius of 35 μm, then the averaged pressure increase would be (1050/35)^2^ ⋅ 0.1 GPa = 90 GPa. Note that in RDAC, the maximum pressure at the center drastically grows even at constant *σ*_*n*_ (Fig. 4) due to pressure redistribution along the radius.

Since our physical model does not contain any length-scale parameters, the same effects are expected for geometrically similar configurations for larger volume anvils. This is true for smaller pressures since they are limited by the strength of anvils made of hard alloys. In particular, a high pressure gradient was observed in experiments with large scale rotational Drickamer anvils with flat tips^28^. The pressure gradient can be significantly magnified for beveled anvils and small sample thickness. Note that detailed mechanics treatment of the compression in DAC and compression and torsion in RDAC under megabar pressure are presented in Refs 8 and 33.

## Appendix

### Derivation of a simplified equilibrium equation

**Figure 7:**
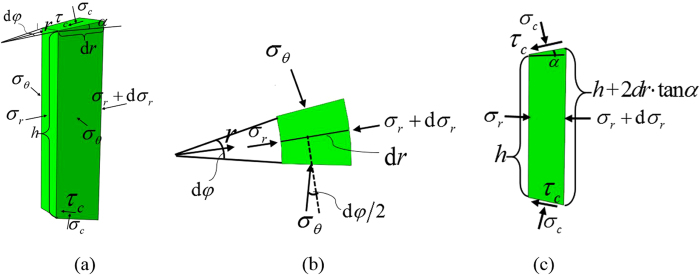
Free-body diagram for derivation of the equilibrium condition in the radial direction for an infinitesimal element of the sample in 3D (**a**), and 2D projections (**b**,**c**).

Let us consider equilibrium in the radial direction of an infinitesimal element of the sample in the deformed configuration in DAC at an arbitrary position *r* shown in Fig. 7(a). All stresses and geometric parameters are presented in Fig. 7, where *σ*_*r*_ and *σ*_*θ*_ are averaged over the sample thickness normal stresses, and *τ*_*c*_ and *σ*_*c*_ are the local friction and normal contact stress at the contact surface between the sample and anvil, respectively. Summation of all forces in the *r* direction yields


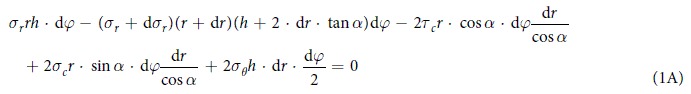


After simple algebra and neglecting the higher order infinitesimal terms, we obtain the differential equilibrium equation


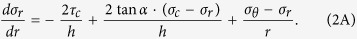


### A complete system of equations for large-strain elastoplasticity for a sample

We designate contractions of the second-order tensors ***A*** = {*A*_*ij*_} and ***B*** = {*B*_*ij*_}over one and two indices as ***A*** ⋅ ***B*** = {*A*_*ij*_*B*_*jk*_} and ***A***:***B*** = {*A*_*ij*_*B*_*ji*_}, respectively. Similarly for the fourth-order tensors ***A*** and ***B***, contractions over one and two indices are defined as ***A*** · ***B*** = {*A*_*ijkm*_*B*_*mnlq*_}and ***A***:***B*** = {*A*_*ijkm*_*B*_*mklq*_}. The subscripts *s* and *a* mean symmetrization and anti-symmetrization, respectively. The superscripts *t* and −1 designate the transposition and inverse of a tensor. The subscripts *e* and *p* mean elastic and plastic deformation gradient or strains, and ***I***is the second-order unit tensor. Complete system of equations presented below can be found in ref. 8.

#### Kinematics

The motion of material with large elastic and plastic deformations is described by a vector function ***r*** = ***r***(***r***_0_, *t*). Here, ***r***_0_ and ***r*** are the position vectors of material points in the reference configuration Ω_0_ at the instant *t*_0_ and in the actual configuration Ω at time instant *t*. The deformation gradient


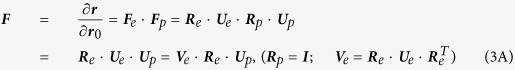


is decomposed into elastic ***F***_*e*_ and plastic ***F***_*p*_ contributions, where ***F***_*p*_ is the deformation gradient obtained after a complete release of stresses in the local vicinity of each material point. In addition, ***U***_*e*_ and ***U***_*p*_ are the symmetric elastic and plastic right stretch tensors, ***V***_*e*_ is the elastic left stretch tensor, and ***R***_*e*_ and ***R***_*p*_ are the proper orthogonal elastic and plastic rotation tensors.

Eulerian and Lagrangian elastic strain tensors are


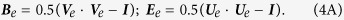


Decomposition of the velocity gradient 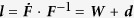 into symmetric deformation rate ***d*** = (***l***)_*s*_ and antisymmetric spin ***W*** = (***l***)_*a*_ in combination with Eq. (3A) results in the following decomposition of the deformation rate into elastic and plastic contributions:


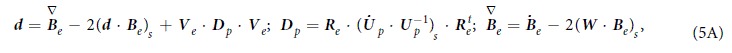


where ***D***_*p*_ is the plastic deformation rate, and 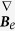 is the Jaumann objective time derivative.

#### Elasticity rule

The following isotropic nonlinear elastic rule yields


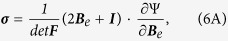


where ***σ*** is Cauchy stress, *det **F*** is the determinate of the tensor ***F***, and the most popular elastic potential for high pressure Ψ is the third-order Murnaghan potential^29^:


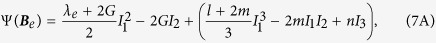


where *λ*_*e*_, *G, m, l*, and *n* are material parameters; *I*_1_, *I*_2_, and *I*_3_, are the first, second and third invariants of the strain tensor ***B***_*e*_:


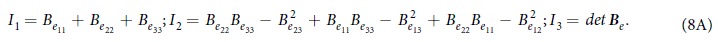


#### Plasticity

The simplest pressure-dependent J_2_ flow theory is used:


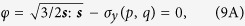


where *σ*_*y*_ is the yield strength, *p* is the hydrostatic pressure, and *q* is the accumulated plastic strain. Then the plastic flow rule is


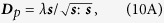


where ***s*** is the deviatoric stress, and *λ*(*λ* > 0) is a scalar function determined from the consistency condition 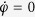. Levitas^14^ found for more than 60 materials belonging to different classes (*e.g*., metals, alloys, rocks, oxides, compacted powders) that, despite the strain-induced anisotropy and history-dependence, above some level of plastic strain and for a deformation path without sharp changes in directions (monotonous deformation), the initially-isotropic polycrystalline materials are deformed as a perfectly plastic and isotropic material with a strain history-independent limiting surface of the perfect plasticity. These results lead to the exclusion of *q* from the relationship for the yield strength in Eq. (6A), *σ*_*y*_(*p, q*) = *σ*_*y*_(*p*). The linear dependence of yield strength on pressure *p* is accepted in this paper


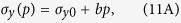


where 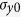 is the initial yield strength at pressure *p* = 0 and *b* is a parameter. The equilibrium equations have standard form


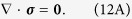


#### Material parameters

Rhenium has been of particular interest due to its large bulk modulus (*K*), shear modulus (*G*), and high strength^16^,^30^. The following properties of rhenium are used in simulations:

Elastic constants^8^,^16^,^30^


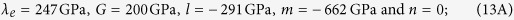


Plastic constants^8^,^16^


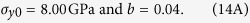


### Nonlinear anisotropic elasticity for single-crystal diamond

The traditional elasticity rule has the form


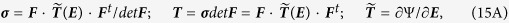


where 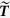 is the second Piola-Kirchhoff stress and ***T*** is the Kirchhoff stress. Since there is no plastic deformation in a diamond, the subscript *e* is dropped. Under very high pressure, it is necessary to consider at least the third-order potential Ψ consistent with the cubic symmetry:


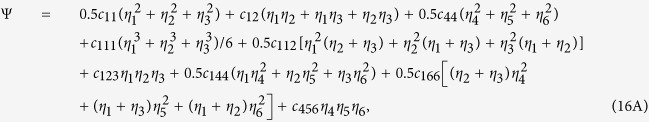


where *η*_1_ = *E*_11_, *η*_2_ = *E*_22_, *η*_3_ = *E*_33_, *η*_4_ = 2*E*_23_, *η*_5_ = 2*E*_31_, and *η*_6_ = 2*E*_12_.

In this paper, the second-order elastic constants are used as ref. 31:


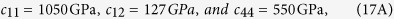


and the third-order elastic constants are used as ref. 32:


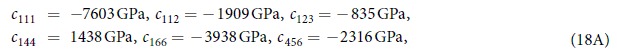


In simulations^8^, the friction coefficient in the Coulomb friction rule is *μ* = 0.1.

## Additional Information

**How to cite this article:** Feng, B. and Levitas, V. I. Pressure Self-focusing Effect and Novel Methods for Increasing the Maximum Pressure in Traditional and Rotational Diamond Anvil Cells. *Sci. Rep.* **7**, 45461; doi: 10.1038/srep45461 (2017).

**Publisher's note:** Springer Nature remains neutral with regard to jurisdictional claims in published maps and institutional affiliations.
